# Disease patterns and specific trajectories of anti-MDA5-related disease: a multicentre retrospective study of 70 adult patients

**DOI:** 10.3389/fimmu.2023.1319957

**Published:** 2024-01-08

**Authors:** Hubert de Boysson, Marie Cuchet, Charles Cassius, Pierre Cuchet, Christian Agard, Alexandra Audemard-Verger, Sylvain Marchand-Adam, Raphaëlla Cohen-Sors, Laure Gallay, Julie Graveleau, Cécile Lesort, Kim Ly, Alain Meyer, Grégoire Monseau, Antoine Néel, Bernard Bonnotte, Laurent Pérard, Nicolas Schleinitz, Delphine Mariotte, Brigitte Le Mauff, Gwladys Bourdenet, Wafa Masmoudi, Samuel Deshayes, Anaël Dumont, Anne Dompmartin, Diane Kottler, Achille Aouba

**Affiliations:** ^1^ Department of Internal Medicine, Caen University Hospital, Caen, France; ^2^ Department of Dermatology, Caen University Hospital, Caen, France; ^3^ Department of Dermatology, France Saint Louis Hospital, (AP-HP), Paris, France; ^4^ Department of Pneumology, Caen University Hospital, Caen, France; ^5^ Nantes Université, Centre Hospitalier et Universitaire (CHU) Nantes, Service de Médecine Interne, Nantes, France; ^6^ Department of Internal Medicine, Tours University Hospital, Tours, France; ^7^ Department of Pneumology, Tours University Hospital, Tours, France; ^8^ Department of Dermatology, Amiens University Hospital, Amiens, France; ^9^ Service de Médecine Interne et Immunologie Clinique, Hôpital Édouard Herriot, Hospices Civils de Lyon, Lyon, France; ^10^ Department of Internal Medicine, Saint-Nazaire Hospital, Saint-Nazaire, France; ^11^ Department of Dermatology, Edouard Herriot Hospital, Hospices civiles de Lyon (HCL), Lyon, France; ^12^ Department of Internal Medicine, Limoges University Hospital, Limoges, France; ^13^ Department of Rheumatology, Strasbourg University Hospital, Strasbourg, France; ^14^ Department of Intensive Medicine, Poitiers University Hospital Center, Poitiers, France; ^15^ Department of Internal Medicine, Dijon University Hospital, Dijon, France; ^16^ Department of Internal Medicine, Saint Joseph Saint Luc Hospital, Lyon, France; ^17^ Department of Internal Medicine, La Timone University Hospital, Assistance Publique - Hopitaux de Marseille (AP-HM), Marseille, France; ^18^ Department of Immunology, Caen University Hospital, Caen, France; ^19^ Department of Immunology, Amiens University Hospital, Amiens, France; ^20^ HEMATIM – EA4666, Jules Verne University of Picardie, Amiens, France; ^21^ Department of Dermatology, Rouen University Hospital, Rouen, France

**Keywords:** anti-MDA5 dermatomyositis, prognosis, rapidly progressive interstitial lung disease, thromboembolic events, malignancy

## Abstract

**Introduction:**

This study aimed to provide an updated analysis of the different prognostic trajectories of patients with anti-melanoma differentiation-associated gene 5 (MDA5) antibodies.

**Methods:**

Among a cohort of 70 patients, baseline characteristics and phenotypes, treatments and outcomes were analyzed. A Cox proportional hazards model was used to identify factors associated with poor outcomes, i.e., death or progressive disease at the last follow-up.

**Results:**

Among the 70 patients, 45 were women, and 54 were Caucasian. A dermatologic involvement was observed in 58 (83%) patients, including 40 with MDA5 vasculopathy-related skin lesions. Muscular involvement was observed in 39 (56%) patients. Interstitial lung disease (ILD) was observed at baseline in 52 (74%) patients, including 23 (44%) who developed rapidly progressive (RP) ILD. Seven (10%) patients showed thromboembolic complications within the first weeks of diagnosis, and eight (11%) other patients developed a malignancy (4 before the diagnosis of anti-MDA5 disease). Poor outcomes were observed in 28 (40%) patients, including 13 (19%) deaths. Among the 23 patients with RP-ILD, 19 (79%) showed poor outcomes, including 12 (63%) who died. In multivariate analyses, RP-ILD (hazard ratio (HR), 95% CI: 8.24 [3.21–22], p<0.0001), the occurrence of thromboembolic events (HR: 5.22 [1.61–14.77], p=0.008) and the presence of any malignancy (HR: 19.73 [6.67–60], p<0.0001) were the three factors independently associated with poor outcomes.

**Discussion:**

This new independent cohort confirms the presence of different clinical phenotypes of anti-MDA5 diseases at baseline and the poor prognosis associated with RP-ILD. Thromboembolic events and malignancies were also identified as prognostic factors.

## 1 Introduction

Dermatomyositis (DM) is one of the subgroups of inflammatory myopathies (1). Myositis-specific autoantibodies are currently used to identify the different clinical phenotypes of DM that often share some common findings, such as cutaneous, muscular, articular and pulmonary tropism. Anti-melanoma differentiation-associated gene 5 (MDA5) antibodies were identified in 2005 in a subset of Japanese patients with amyopathic DM and rapidly progressive interstitial lung disease (RP-ILD) (2). Additional studies demonstrated that 17 to 100% of patients with anti-MDA5 DM had amyopathic forms (3). Among the different clinical forms of DM, anti-MDA5 DM more commonly affects women and is more prevalent in the Asian population (11 to 60% of DM) than in the Caucasian population (7 to 16% of DM) (3).

In addition to the minimal or absent muscle involvement, the high prevalence of RP-ILD and the presence of ulcerative skin lesions are probably the most representative hallmarks of anti-MDA5 DM (3). In the main Asian cohorts, more than 80% of patients with anti-MDA5 DM develop ILD, with rapid progression in 39 to 100% (2, 4–11). Less is known about Caucasian patients, but the few existing cohorts report a lower rate of ILD, close to 60% of patients (12–17). Most of these studies highlighted the high mortality rate of patients with RP-ILD, often within the first year (2, 4–18).

In 2020, Allenbach et al. (18), in a French cohort study of 83 anti-MDA5 DM patients, identified three patient clusters according to their specific and distinct clinical phenotypes, with different related prognoses. The first phenotype has a predominance of women with RP-ILD and carries the worst prognosis, with a mortality rate near 80%. Patients in the second cluster mainly demonstrate cutaneous and articular involvement, with few cases of RP-ILD (<20%), conferring a better prognosis. Finally, the last subgroup mainly includes men with severe skin vasculopathies and frequent signs of myositis; RP-ILD affects a quarter of this group, and they exhibit a prognosis of intermediate severity (18).

Although none of these findings have been replicated, a high ferritin level at diagnosis (6), high anti-MDA-5 antibody levels (4), older age and periungual erythema (11) were identified as predictive factors of a poor prognosis and/or RP ILD development. In contrast with some other forms of DM, such as anti-TIF1γ (anti-transcription intermediary factor 1-gamma) DM, anti-MDA5 DM does not seem to be significantly associated with an increased risk of cancer (3, 4, 7, 9, 10). In addition, based on the information described in the published studies, some important issues remain, especially regarding treatments and outcomes. The recent identification of specific disease patterns may open the way to different therapeutic strategies.

Taken together, we aimed to confront this new independent cohort of anti-MDA5 DM patients to that of Allenbach et al. (18), with the following objectives: 1) to determine whether it was possible in a real-life setting to include patients into a distinct cluster subgroup based on the work-up obtained at baseline and during follow-up; 2) to analyze outcomes according to the different treatment regimens; 3) to eventually identify new factors associated with poor outcomes.

## 2 Patients and method

### 2.1 Patient selection

This retrospective multicentre study included patients from 14 French hospitals, different from those involved in the previous cohort published in 2020 (18). Physicians from the departments of Internal Medicine and Clinical Immunology, Dermatology and Pneumology were directly asked to participate and include their patients with anti-MDA5 disease diagnosed between January 2008 and August 2020.

We included patients satisfying the two following criteria: 1) positivity for MDA5 antibodies and 2) the presence of clinical manifestations considered linked to the presence of anti-MDA5 antibodies. Since we aimed to provide an overview of disease associated with anti-MDA5 antibodies, we did not consider mandatory the presence of cutaneous or muscular findings.

We excluded patients under 16 years old.

In the different centres, anti-MDA5 antibody detection was performed by line immunoassays using recombinant anti-MDA5 antigen (Euroimmun [Germany] or D-Tek [Belgium]) according to the manufacturer’s protocol. Only moderate or strong reactivity results were considered in the present study.

This study was conducted in accordance with the Declaration of Helsinki and its amendments and was approved by the local institutional review board of Caen University Hospital (*CLERS N°123/2018-12-27*123).

### 2.2 Studied parameters

A standardized dataform was created for this study and was sent to each invited physician. Retrieved information included demographics; clinical manifestations at diagnosis; laboratory parameters; histologic and electrophysiologic results when available; imaging, especially a chest CT scan, the results of echocardiography and respiratory functional exploration (RFE); and the administered treatments and outcomes. We particularly detailed the clinical manifestations associated with anti-MDA5 DM.

Cutaneous manifestations were distributed into 4 subgroups. The first subgroup included findings considered pathognomonic of DM: Gottron’s papules, Gottron’s sign, and heliotrope rash. The second subgroup gathered manifestations considered very suggestive of DM: periungual telangiectasias with dystrophic cuticles; painful periungual erythema; cuticular haemorrhages or small infarcts; V signs, defined by macular violaceous erythema of the neck and the upper chest; shawl signs corresponding to erythema of the nape of the neck, the upper back and the posterior face of the shoulders; and holster signs consisting of scaling erythema of the external surface of the thighs and hips and extensor surface of the upper limbs. The third cutaneous subgroup included findings specific to anti-MDA5 phenotypes: ulcerations that can involve finger pulps or the circumference of the nail; the posterior face of the hands, ears or nose, and/or elbows or knees; palmar papules, and oral ulcers. Finally, the last subgroup included less specific dermatological lesions: poikiloderma, mechanic’s hands, calcinosis panniculitis, erythroderma, photosensitivity, diffuse alopecia, and pruritus.

We applied the classification used in the study of Allenbach et al. (18) to our patients. The classification into the three clusters was based on the clinical presentation and evolution of the patients and was independently made by two investigators (HdB, an internist, and MC, a dermatologist). In discordant cases, consensus was obtained by discussion with another pair of physicians of the same specialties (AA and DK).

The ILD diagnosis was based on high-resolution CT imaging. RP-ILD was defined by a respiratory worsening within three months following the previous respiratory evaluation and the demonstration of an increase in opacities on CT scan and/or a >10% decrease in vital capacity on respiratory function explorations (19).

Disease trajectory was judged on a simplified binary mode, i.e., favourable versus a poor outcome. Favorable outcomes at the last follow-up were defined by disease remission, improvement or stability. Death or disease worsening defined poor outcomes. In patients with dissociated trajectories (e.g., skin improvement but respiratory worsening), a poor outcome was assigned if the worsening involved respiratory or cardiac functions. We then compared patients with good and poor outcomes and aimed to identify factors associated with poor outcomes.

Any malignancy or thromboembolic event was also reported.

### 2.3 Statistical analysis

Categorical variables are expressed as numbers (%), and quantitative variables are expressed as medians [range]. To compare two groups, the categorical variables were analyzed using the Pearson or Fisher chi-square test, as appropriate, and quantitative variables were analyzed using Wilcoxon’s rank-sum test.

A Cox proportional hazards model was used to determine factors predictive of poor outcomes. Hazard ratios (HRs) and 95% confidence intervals (CIs) were computed for each factor in the univariate analysis and in the multivariate model with a backwards stepwise approach using variables that reached p<0.2 in the univariate analyses.

The statistical analyses were computed using JMP 9.0.1 (SAS Institute Inc., Cary, NC, USA). A p ≤ 0.05 defined statistical significance.

## 3 Results

### 3.1 Patient characteristics at baseline

The cohort included 70 patients exhibiting anti-MDA5 antibodies associated with clinical manifestations. Among them, 45 (64%) were women and 54 (77%) were of Caucasian origin. The median age at disease onset was 57 [16–84] years old. Their baseline characteristics are described in **Table 1**.

**Table 1:** Baseline characteristics of 70 patients with anti-MDA5 antibodies.

	All (n=70)
Demographics	
Female	45 (64)
Age at diagnosis	57 [16–84]
Caucasian origin	54 (77)
Asian origin	3 (4)
Other origins	13 (19)
Clinical manifestations at diagnosis	
Altered health status	54 (77)
Fever	17 (25)
Raynaud’s phenomenon	14 (20)
Arthralgia/Arthritis	40 (57)
Cutaneous involvement	58 (83)
Myalgias	31 (44)
ENT/swallowing troubles	19 (27)
Dyspnea	37 (53)
Digestive symptoms	11 (16)
Thromboembolic event	7 (10)
Subgroup distinction	
Cluster 1	15 (21)
Cluster 2	44 (63)
Cluster 3	11 (16)
Laboratory work-up	
Creatine kinase, UI/l	104 [20–13742]
Ferritin level, µg/l	570 [37–10436]
C-reactive protein, mg/l	5 [0–175]
LDH, UI/l	476 [136–1271]
Muscular work-up	
Abnormal electromyoneurography	9/31 (29)
Abnormal muscular MRI	8/10 (80)
Abnormal muscular biopsy	12/14 (86)
Cardio-respiratory explorations	
Abnormal RFE	18/49 (37)
ILD on first CT-scan	52 (74)
PAH on echocardiography	4 (6)

Values are displayed as numbers (%) or medians [range]. ENT, ear, nose & throat; LDH, lactate dehydrogenase; MRI, magnetic resonance imaging; RFE, respiratory functional exploration; ILD, interstitial lung disease; CT scan, computed tomography scan; PAH, pulmonary arterial hypertension.

Cluster 1 included patients with rapidly progressive interstitial lung disease, Cluster 2 included patients with predominant cutaneoarticular presentation, and Cluster 3 included patients with mainly a vasculo-myositic presentation.

Dermatological involvement was observed in 58 (83%) patients, and the different cutaneous and mucosal manifestations are detailed in **Table 2**. Among these 58 patients, 56 (97%) showed some lesions compatible with DM, and 51 (88%) exhibited pathognomonic DM lesions. Specific vasculitic lesions of the anti-MDA5 phenotype were found in 40 (69%) patients, mainly acral ulcerations in 33 (57%).

**Table 2:** Details of the clinical findings in the 58/70 patients with anti-MDA5-related mucocutaneous involvement.

Mucocutaneous features	Patients with skin and mucosal involvement (n=58)
**Pathognomonic of DM**	51 (88)
Gottron’s papules	40 (69)
Heliotrope rash	31 (53)
Gottron’s sign	22 (38)
**Suggestive of DM**	56 (97)
Scalp erythema	9 (16)
Face erythema	33 (57)
V area erythema (V sign)	25 (43)
Periungual erythema	23 (40)
Shawl’s sign	12 (21)
Hips and thighs erythema (holster sign)	7 (12)
Flagellate erythema	4 (7)
**Specific of Anti MDA5 phenotype**	40 (69)
Ulcerations:	33 (57)
Finger pulp	24 (41)
Periungual	23 (33)
Dorsal hands	9 (16)
Ear or nose	8 (14)
Elbows or knees	6 (10)
Palmar papules	19 (33)
Oral pain/	16 (28)
**Less specific lesions**	36 (63)
Mechanic’s hands	16 (28)
Photosensitivity	16 (28)
Ocular sicca syndrome	14 (24)
Alopecia	10 (17)
Pruritus	8 (14)
Poikilodermia	4 (7)
Calcinosis cutis	4 (7)
Panniculitis	2 (3)
Vesiculo-bullous lesions	1 (2)
Erythroderma	2 (3)

Values are displayed as numbers (%).

Myalgias were present at diagnosis in 31 (44%) patients, including 12 (i.e., 17% of the overall cohort) who exhibited an increased level of CPK (median level at 1176 [334–13742] U/l). Three additional patients had increased CPK levels, although they did not have muscular symptoms. Notably, among the 31 patients who underwent electromyoneurography, 9 (29%) had abnormal findings suggestive of a myogenic syndrome. Based on the myalgias, CPK increase and/or a pathological electromyography work-up, 39 (56%) patients were considered to have specific muscular involvement at baseline.

Dyspnea was described at diagnosis in 37 (53%) patients, including 34 (92% of those with dyspnea and 49% of the whole cohort) who had concomitant signs of ILD on CT scan. Eighteen additional patients had ILD on CT scan, although they did not complain of dyspnea. Altogether, at baseline, 52 (74%) of the patients with anti-MDA5 antibodies showed typical features of ILD on CT scan. RFE was performed at baseline in 49 patients, including 39 with ILD on CT scan and 10 without ILD. Abnormal findings were observed in 17 (44%) out of the 39 patients with ILD and in one (10%) of the patients without ILD on CT scan. Four patients with ILD had concomitant signs of pulmonary arterial hypertension on echocardiography.

Articular involvement and Raynaud’s phenomenon were described at diagnosis in 40 (57%) and 14 (20%) patients, respectively. In addition, 7 (10%) patients exhibited thromboembolic events within the first weeks following the diagnosis.

Based on the clusters described by Allenbach et al. [18], we identified 15 (21%), 44 (63%) and 11 (16%) patients corresponding to the predominant RP-ILD Cluster 1, cutaneo-articular Cluster 2 and vasculo-myositis Cluster 3, respectively. The comparison of the 3 clusters’ characteristics (**Table 3**) showed that 6 items were significantly different among the three groups, namely, the age at diagnosis, frequencies of Raynaud phenomenon, joint involvement, cutaneous involvement, ILD on the first CT scan and abnormal RFE. No difference was observed among the 3 clusters regarding initial laboratory parameters.

**Table 3:** Baseline characteristics of 70 patients with anti-MDA5 antibodies according to the three distinct subgroups.

	Cluster 1 (n=15)	Cluster 2 (n=44)	Cluster 3 (n=11)	*P*
Demographics				
Female	11 (73)	30 (68)	4 (36)	0.1
Age at diagnosis	66 [45–79]	56 [16–80]	45 [26–84]	0.04
Clinical manifestations at diagnosis				
Altered health status	9 (60)	34 (77)	11 (100)	0.06
Fever	5 (33)	9 (20)	3 (17)	0.59
Raynaud’s phenomenon	0	7 (16)	7 (63)	0.0002
Arthralgia/Arthritis	2 (13)	33 (75)	5 (45)	0.0001
Cutaneous involvement	7 (47)	42 (95)	9 (82)	<0.0001
Signs of MDA5-associated vasculopathy	5 (33)	27 (61)	8 (73)	0.09
Muscular symptoms	3 (20)	21 (48)	7 (64)	0.07
ENT/swallowing troubles	2 (13)	15 (34)	2 (18)	0.23
Dyspnea				
Digestive symptoms	2 (13)	7 (16)	2 (18)	0.94
Thromboembolic event	2 (13)	4 (9)	1 (9)	0.89
Laboratory work-up				
Creatine kinase, UI/l	70 [20–1252]	105 [20–13742]	224 [20–3500]	0.24
Ferritin level, µg/l	1374 [46–10436]	528 [79–5800]	571 [79–3067]	0.64
LDH, UI/l	603 [190–787]	436 [178–1271]	653 [136–896]	0.34
Muscular work-up				
Abnormal electromyoneurography	1/5 (20)	7/21 (33)	1/5 (20)	0.75
Abnormal muscular MRI	0	7/9 (78)	1/1 (100)	0.6
Abnormal muscular biopsy	2/3 (67)	7/7 (100)	3/4 (75)	0.30
Cardio-respiratory explorations				
Abnormal RFE	6/11 (55)	7/30 (23)	5/8 (63)	0.047
ILD on first CT-scan	15/15 (100)	28 (64)	9 (82)	0.02

Values are displayed as numbers (%) or medians [range]. ENT, ear, nose & throat; LDH, lactate dehydrogenase; MRI, magnetic resonance imaging; RFE, respiratory functional exploration; ILD, interstitial lung disease; CT-scan, computed tomography scan; PAH, pulmonary arterial hypertension. Cluster 1 included patients with rapidly progressive interstitial lung disease, Cluster 2 included patients with predominant cutaneoarticular presentation, and Cluster 3 included patients with mainly vasculo-myositic presentation.

Among the 70 patients, positive antinuclear antibodies were found in 38 (54%) patients, of which 7 and 3 were anti-SSA (Ro-60) and anti-Ro52 specific, respectively. Three had anti-TIF1-γ antibodies.

### 3.2 Treatments and outcomes

Except for five patients, all patients received glucocorticoids. Four patients only showed cutaneous manifestations that were treated with topical corticosteroids and hydroxychloroquine. The fifth patient, who exhibited a non-rapidly progressive ILD and a slight muscular involvement, had a contraindication for glucocorticoids and was therefore treated with intravenous immunoglobulins and cyclophosphamide, followed by mycophenolate mofetil. He was in remission at the last follow-up visit. The other immunomodulatory/immunosuppressive strategies included various combinations of hydroxychloroquine, mycophenolate mofetil, azathioprine, plasma exchanges, methotrexate, intravenous immunoglobulins, rituximab and/or cyclophosphamide (**Table 4**).

**Table 4:** Baseline characteristics, treatments and outcomes of anti-MDA5 patients according to the disease status at last follow-up.

	Stability or remission (n=42; 60%)	Worsening or death (n=28; 40%)	*P*
Demographics			
Female	26 (62)	19 (68)	0.80
Age <55 years	20 (48)	11 (39)	0.49
Clinical findings at diagnosis			
Altered health status	33 (79)	21 (75)	0.73
Fever	8 (19)	9 (32)	0.21
Raynaud’s phenomenon	11 (26)	3 (11)	0.14
Skin and mucosal involvement	35 (83)	23 (82)	0.90
Signs of MDA5-associated vasculopathy	24 (57)	16 (57)	1
Arthralgia/Arthritis	24 (57)	16 (57)	1
Myalgias	21 (50)	10 (36)	0.24
ENT/swallowing troubles	10 (24)	9 (32)	0.44
Dyspnea	22 (52)	15 (54)	0.92
Digestive symptoms	7 (17)	4 (14)	1
Thromboembolic event	2 (5)	5 (18)	0.11
Creatine kinase (UI/L) level at baseline	121 [20–13742]	70 [20–3373]	0.1
Ferritin level at baseline, µg/l	571 [58–5800]	568 [37–10436]	0.66
LDH level at baseline, UI/1	472 [136–1271]	480 [178–834]	0.64
Cardio-respiratory explorations			
Abnormal RFE	11/28 (39)	7/21 (33)	0.77
ILD on first CT-scan	28 (67)	24 (86)	0.1
PAH on echocardiography	0	4 (14)	0.02
Treatments received			
Hydroxychloroquine	15 (36)	6 (21)	0.29
Glucocorticoids	38 (91)	27 (96)	0.64
Mycophenolate mofetil	12 (29)	8 (29)	1
Azathioprine	10 (24)	2 (7)	0.11
Plasma exchanges	2 (5)	5 (18)	0.11
Methotrexate	15 (36)	9 (32)	0.80
Intravenous immunoglobulins	20 (48)	11 (39)	0.49
Rituximab	5 (12)	4 (14)	1
Cyclophosphamide	11 (26)	10 (36)	0.39
Total follow-up (months)	25 [1–147]	12 [0.1–58]	<0.001
Cancer at any time	0	8 (29)	0.0003

Values are displayed as numbers (%) or medians [range]. ENT, ear, nose & throat; RFE, respiratory functional exploration; ILD, interstitial lung disease; CT scan, computed tomography scan; PAH, pulmonary arterial hypertension.

**Figure 1:**
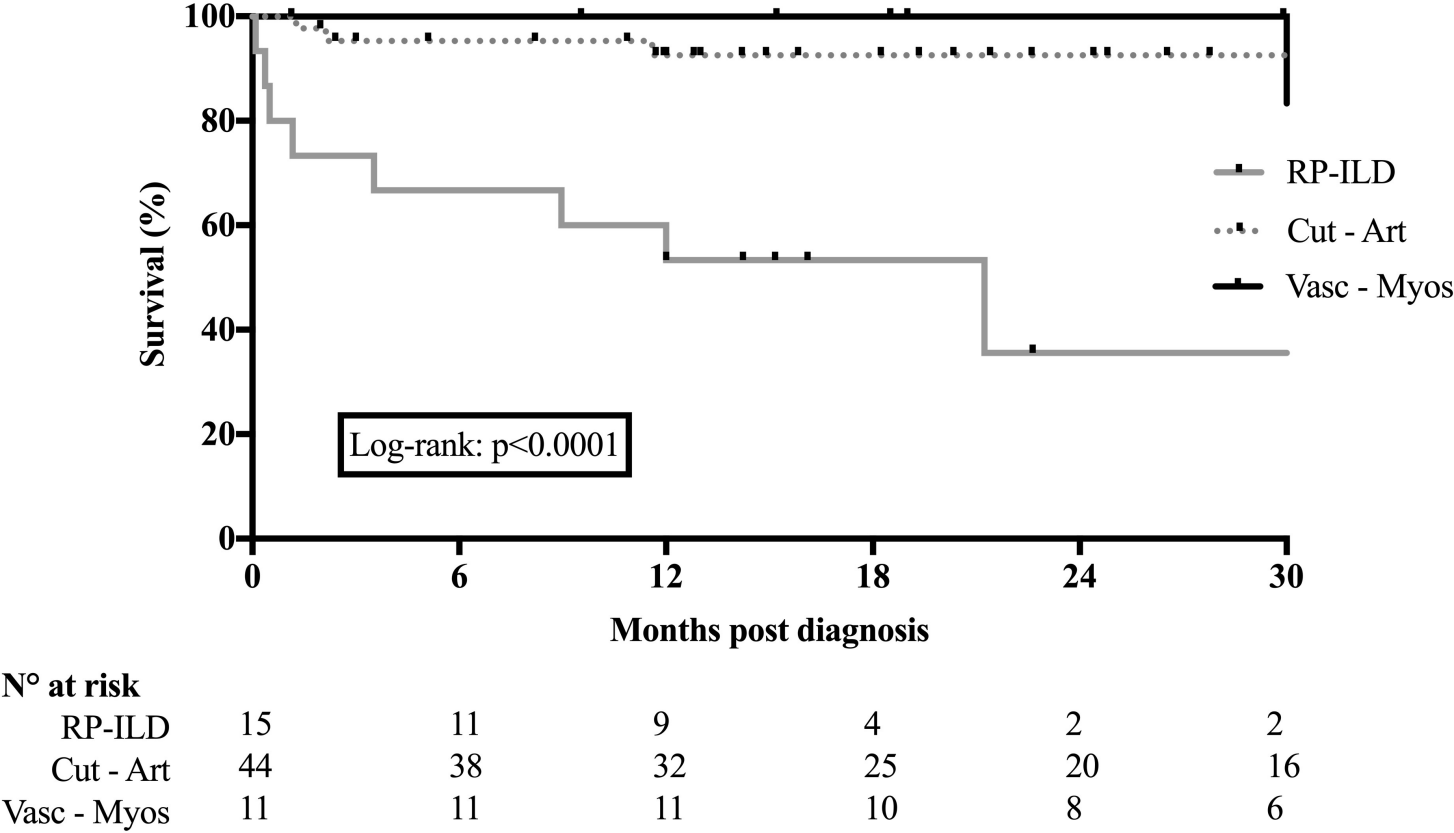
Survival of patients with anti-MDA5 antibodies according to the 3 initial main phenotypes. Main phenotypes are predominant rapidly progressive interstitial lung disease, predominant cutaneoarticular presentation and predominant vascular-myositic presentation.

After a median follow-up of 18 [0.1—147] months, 42 (60%) and 28 (40%) of the patients showed favorable and poor outcomes, respectively. Poor outcomes were observed in 11/15 (73%) patients from Cluster 1, in 15/44 (34%) patients from Cluster 2 and 2/11 (18%) patients from Cluster 3 (p=0.008). Of the 52 patients with ILD, 23 (44%) developed RP-ILD, 15 were in the Cluster 1, and 6 and 2 in the Cluster 2 and 3, respectively. Poor outcomes were observed in 19 (79%) patients with RP-ILD, including 12 (63%) who died from RP-ILD. An additional patient in the Cluster 2 died from an infection. Survival curves are shown in **Figure 1** (log-rank: p<0.0001). Of note, 2 of the 3 patients with anti-Ro52 antibodies showed poor respiratory outcomes.

Eight (11%, 2 in Cluster 1, 6 in Cluster 2) patients developed a malignancy (3 myeloproliferative disorders, one lymphoma, one breast cancer, one ovarian cancer, one bronchial cancer and one thyroid cancer), including one of the three patients who exhibited anti-TIF1-γ antibodies. The diagnosis of malignancy preceded and followed the diagnosis of MDA-related DM in 4 and 4 patients, respectively. All 8 patients had poor outcomes (5 died with RP-ILD, and 3 showed disease progression at the last follow-up). The 7 patients who developed thromboembolic events did not have any concomitant malignancies. Five of them had poor outcomes, including 3 who died from RP-ILD.

Ferritin or LDH levels at baseline, as well as therapeutic management, were not different between patients with favorable and poor outcomes (**Table 4**).

### 3.3 Factors associated with poor outcomes

Using a Cox proportional hazards model, multivariate analyses showed that RP-ILD (hazard ratio (HR), 95% CI: 8.24 [3.21–22], p<0.0001), the occurrence of thromboembolic events (HR: 5.22 [1.61–14.77], p=0.008) and the presence of any malignancy (HR: 19.73 [6.67–60], p<0.0001) were the three factors independently associated with a risk of poor outcomes (**Table 5**).

**Table 5:** Baseline factors associated with worsening or death in patients with anti-MDA5-related dermatomyositis in a Cox proportional hazards model.

	Univariate HR [95% CI]	p	Multivariate HR [95%CI]	p
RP-ILD	4.38 [1.92—9.79]	0.0007	8.24 [3.21–22]	<0.0001
Face erythema	0.53 [0.23—1.16]	0.11		
Hands erythema	0.43 [0.13—1.12]	0.09		
Periungual erythema	0.35 [0.12–0.86]	0.02		
Raynaud phenomenon	0.35 [0.08–1.01]	0.05		
Muscular symptoms	0.53 [0.23–1.13]	0.1		
Alveolar opacities on CT scan	2 [0.91–4.66]	0.08		
PAHT	4 [1.17–10.65]	0.03		
Thromboembolic event at diagnosis	2.90 [0.97—7.11]	0.06	5.22 [1.61–14.77]	0.008
Any malignancy	5.81 [2.38—12.91]	0.0003	19.73 [6.67–60]	<0.0001
Azathioprine use	0.20 [0.03—0.71]	0.009	0.18 [0.03–0.70]	0.01
Hydroxychloroquine use	0.51 [0.19—1.20]	0.13		

CI, confidence interval; HR, hazards ratio; RP-ILD, rapidly progressive interstitial lung disease; PAHT, pulmonary arterial hypertension.

Conversely, azathioprine use showed a protective effect (HR: 0.18 [0.03–0.70], p=0.01). **Supplementary Table S1** compares patients with and without azathioprine. Except for a longer follow-up duration among patients who received azathioprine (p=0.0009), we did not observe any differences between baseline characteristics, initial paraclinical work-up, treatments received and death rate. Of note, 9/12 (75%) patients who received azathioprine were in Cluster 2.

## 4 Discussion

Beyond the relevant prognostic distinction of anti-MDA patients into the three subgroups previously described (18), this study identified some previously unknown comorbidities and treatment approaches influencing disease prognosis. Interestingly, some of them probably appeared unrelated to the underlying MDA5-related disease. Indeed, in addition to RP-ILD, our study demonstrated that thromboembolic events within the first weeks of MDA5-related disease or a recent history or occurrence of malignancies were also significantly more frequently observed in patients with poor outcomes, regardless of cluster type. In addition, our study identified a possible protective effect of azathioprine. Other para-clinical parameters, including iconographic, electrophysiological and laboratory findings, especially muscle enzymes or ferritin levels, were not found to influence the patient’s outcome.

In this study, we replicated the patients’ subgrouping proposed by Allenbach et al. (18) with the objective of confirming or challenging the relevance of this distinction. We found similar results. More than half of our patients exhibited the cutaneo-articular disease form (Cluster 2) with a better prognosis, and patients with RP-ILD (especially from Cluster 1) showed the worst prognosis.

Based on the poor outcomes associated with the development of ILD, two recent multicentre retrospective studies proposed classifying patients according to three phenotypes or clusters according to the risk of developing RP-ILD (17, 20). Regardless of the disease subgrouping, each cohort study provides similar warning signals regarding the poor outcomes of anti-MDA5-positive patients with ILD. The best therapeutic management of these patients remains unknown, but in practice, it often relies on a combination of glucocorticoids and an intravenous immunosuppressant. More recently, successful lung transplantation has been reported, generally preceded by extracorporeal life support, in patients with RP-ILD (21).

Our result regarding the protective effect of azathioprine should be interpreted with caution since three-fourths of the patients who received this treatment belonged to the cutaneo-articular form of the disease. No other cohort studies reported such results, and replication is needed before any conclusion can be made.

Our study pointed to the presence of a malignancy in 11% of our patients, which is similar to the Spanish cohort (17). Our multivariate analysis indicated the worst prognosis in patients with a concomitant malignancy, but the poor outcomes observed in these patients were linked to MDA5 disease and not cancer evolution. Two-thirds of patients with concomitant malignancy died from RP-ILD. In contrast to the anti-TIF1-γ- or NXP2 (nuclear matrix protein 2)-related myopathies that are known to be associated with malignancies (22, 23), the association of anti-MDA5 disease with cancer has only been reported in a few case reports (3). Another study suggested that the risk of malignancy in anti-MDA5 DM was the same as that in the general population (24). However, the recent Spanish and present cohorts may suggest a more frequent association of both diseases and might invite the inclusion of an initial neoplastic work-up (17). Similarly, to our knowledge, thromboembolic events have not yet been specifically associated with poor outcomes in anti-MDA5-related diseases. We did not observe any association between malignancies and thromboembolic events in our cohort.

Thus, the negative impacts of malignancies and thromboembolic events on patient outcomes appeared as additional comorbidities affecting the general health status of anti-MDA-5 patients. Some hypothetical explanations might be proposed, such as the possible nonoptimal treatment with immunosuppressants in patients with malignancies or the possible more severe presentation requiring a long confinement to bed in patients with thromboembolic events.

This study is impacted by certain limitations, mainly related to its retrospective and unsystematic case-collection design. Indeed, some useful information was not included in the standardized data form sent to each physician in order to favor their participation. Details on the treatments’ combinations and chronologies are lacking. In patients with poor respiratory outcomes, we did not retrieve details about the precise mechanisms involved. In addition, excluding the MDA5 positivity that was checked in all patients, data regarding other immunological assays may be lacking in our patients, especially regarding anti-Ro52 positivity which is associated with poor outcomes in RP-ILD patients (25). Since the initial work-up of patients with anti-MDA5 antibodies was not standardized in our study, some patients with an initial normal chest CT-scan and/or without dyspnea did not undergo RFE. The inclusion of patients into the three distinct clusters might be biased in some patients since an overlapping presentation can exist. In addition, the absence of some important results such as RFE and/or muscular MRI at baseline may have biased the precise clustering of some patients. However, we aimed to provide a distinctive picture of MDA-5 diseases, and this practical and phenotypic clustering was appropriate to distinguish patients. Cluster samples were too small to allow statistical analysis aiming to identify factors associated with the development of RP-ILD.

The real impact of cancers and thromboembolic events remains unclear. However, this study with this purposely chosen design allows for comparison of its data to those of the pioneering French study (18) that established the clustering of this rare disease, which more rarely involves Caucasian populations.

To conclude, this second French study on a different and independent cohort validates the adequacy and relevance of the recent grouping of anti-MDA-5-related diseases into 3 systemic and prognostic clusters. Beyond the RP-ILD cluster associated with the worst prognosis, our study showed that the occurrence of malignancies and thromboembolic events negatively impacted patient outcomes. Therefore, additional, larger observational or interventional studies to better refine the epidemiology, early diagnosis and management of MDA-5 and these targeted comorbidities are needed to improve the overall prognosis of this rare and heterogeneous systemic disease.

## Data availability statement

The raw data supporting the conclusions of this article will be made available by the authors without undue reservation.

## Ethics statement

This study was approved by the local institutional review board of Caen University Hospital (CLERS N°123/2018-12-27123). The studies were conducted in accordance with the local legislation and institutional requirements. The ethics committee/institutional review board waived the requirement of written informed consent for participation from the participants or the participants’ legal guardians/next of kin because the retrospective nature of the study allows according to the French laws to conduct this kind of study.

## Author contributions

HB: Conceptualization, Formal Analysis, Investigation, Methodology, Supervision, Validation, Writing – original draft, Writing – review & editing. MC: Conceptualization, Data curation, Validation, Writing – original draft, Writing – review & editing. CC: Data curation, Validation, Writing – review & editing. PC: Data curation, Validation, Writing – review & editing. CA: Data curation, Validation, Writing – review & editing. AA-V: Data curation, Validation, Writing – review & editing. SM-A: Data curation, Validation, Writing – review & editing. RC-S: Data curation, Validation, Writing – review & editing. LG: Data curation, Validation, Writing – review & editing. JG: Data curation, Validation, Writing – review & editing. CL: Data curation, Validation, Writing – review & editing. KL: Data curation, Validation, Writing – review & editing. AM: Data curation, Validation, Writing – review & editing. GM: Data curation, Validation, Writing – review & editing. AN: Data curation, Validation, Writing – review & editing. BB: Data curation, Validation, Writing – review & editing. LP: Data curation, Validation, Writing – review & editing. NS: Data curation, Validation, Writing – review & editing. DM: Data curation, Validation, Writing – review & editing. BL: Data curation, Validation, Writing – review & editing. GB: Data curation, Validation, Writing – review & editing. WM: Data curation, Validation, Writing – review & editing. SD: Data curation, Validation, Writing – review & editing. ADu: Data curation, Validation, Writing – review & editing. ADo: Data curation, Validation, Writing – review & editing. DK: Data curation, Supervision, Validation, Writing – review & editing. AA: Supervision, Validation, Writing – original draft, Writing – review & editing.
