# Supplementary material for: Disease patterns and specific trajectories of anti-MDA5-related disease: a multicentre retrospective study of 70 adult patients
Source: Front Immunol. 2024 Jan 8;14:1319957. doi: 10.3389/fimmu.2023.1319957 (PMC10800864; doi:10.3389/fimmu.2023.1319957)
Supplement: Supplementary file 1 [file Table_1.docx]

**Supplementary material**

**Supplemental Table S1.** Comparison of patients with anti-MDA5-related disease whether or not they received azathioprine

|  | **Azathioprine (n=12)** | **No azathioprine (n=58)** | **p** |
| --- | --- | --- | --- |
| **Demographics** |  |  |  |
| Female | 9 (75) | 36 (62) | 0.52 |
| Age at diagnosis | 52 [31—70] | 57 [16—84] | 0.67 |
| **Cluster categorization** |  |  | 0.27 |
| Cluster 1 | 1 (8) | 14 (24) | - |
| Cluster 2 | 9 (75) | 30 (52) | - |
| Cluster 3 | 2 (17) | 14 (24) | - |
| **Initial clinical involvements** |  |  |  |
| Initial fever | 1 (8) | 16 (28) | 0.27 |
| Cutaneous | 11 (92) | 47 (81) | 0.68 |
| Raynaud’s phenomenon | 3 (25) | 11 (19) | 0.70 |
| Muscular | 7 (58) | 32 (55) | 1 |
| Arthralgia/Arthritis | 8 (67) | 32 (55) | 0.54 |
| ENT/swallowing troubles | 4 (33) | 15 (26) | 0.72 |
| Dyspnoea | 4 (33) | 33 (57) | 0.20 |
| Digestive | 2 (17) | 9 (16) | 1 |
| Thrombo-embolic event | 1 (8) | 6 (10) | 1 |
| **Follow-up and outcomes** |  |  |  |
| Total follow-up (months) | 52 [2—147] | 16 [0.1—68] | 0.0009 |
| Cancer occurrence | 1 (8) | 7 (12) | 1 |
| Global remission/stability | 10 (83) | 32 (55) | 0.11 |
| Death | 2 (17) | 11 (19) | 1 |

Values are displayed as numbers (%) or medians [range]. Abbreviations: ENT: ear, nose & throat

Cluster 1 included patients with rapidly progressive interstitial lung disease, Cluster 2 included patients with predominant cutaneoarticular presentation, and Cluster 3 included patients with mainly vasculo-myositic presentation.
